# Development, modelling and optimization of process parameters on the tensile strength of aluminum, reinforced with pumice and carbonated coal hybrid composites for brake disc application

**DOI:** 10.1038/s41598-024-67476-x

**Published:** 2024-07-23

**Authors:** Tanimu Kogi Ibrahim, Danjuma Saleh Yawas, Julius Thaddaeus, Bashar Danasabe, Ibrahim Iliyasu, Adetayo Abdulmumin Adebisi, Talib Onimisi Ahmadu

**Affiliations:** 1 Mechanical Engineering Department, Faculty of Engineering, Federal University Wukari, Wukari, Nigeria; 2 Mechanical Engineering Department, Ahmadu Bello University, Zaria, Nigeria; 3 Shell JV Professorial Chair Office, Mechanical Engineering, Ahmadu Bello University, Zaria, Nigeria; 4 Metallurgical and Materials Engineering Department, Ahmadu Bello University, Zaria, Nigeria

**Keywords:** Tensile strength, Stir casting process parameters, Taguchi, Ceramics reinforcement, Al alloy, Mechanical engineering, Materials science

## Abstract

This study focuses on optimizing double stir casting process parameters to enhance the tensile strength of hybrid composites comprising aluminum alloy, brown pumice, and coal ash, intended for brake disc applications. Analytical techniques including X-ray fluorescence, X-ray diffraction, thermogravimetric analysis, and scanning electron microscopy were employed to characterize the composite constituents. The Taguchi method was utilized for experimental design and optimization to determine the optimal weight compositions of brown pumice and coal ash, as well as stir casting parameters (stirrer speed, pouring temperature, and stirring duration). Regression analysis was employed to develop a predictive mathematical model for the tensile strength of the hybrid composites and to assess the significance of process parameters. The optimized composite achieved a predicted tensile strength of 186.81 MPa and an experimental strength of 190.67 MPa using 7.5 vol% brown pumice, 2.5 vol% coal ash, a pouring temperature of 700 °C, stirrer speed of 500 rpm, and stirring duration of 10 min. This represents a 52.23% improvement over the as-cast aluminum alloy’s tensile strength. Characterization results revealed that brown pumice and coal ash contain robust minerals (SiO_2_, Fe_2_O_3_, Al_2_O_3_) suitable for reinforcing metal matrices like aluminum, titanium, and magnesium. Thermogravimetric and differential thermal analyses demonstrated thermal stability up to 614.01 °C for the optimized composite, making it suitable for brake disc applications.

## Introduction

IIn recent years, there has been a growing demand in the automotive industry for high-performance materials capable of enduring extreme conditions while offering superior mechanical properties. In light of this need, researchers have directed their efforts toward the development of innovative materials to enhance the performance of automotive components such as brake discs^1^. The brake disc is a vital component in any vehicle, as the safety and lives of its occupants rely on its effective functioning^2^. Various materials, including grey cast iron, steel, and other metal alloys, have traditionally been used in the production of automotive brake discs. Grey cast iron, in particular, has been favored for its economic viability, high-volume production capabilities, and excellent machinability^3,4^. However, its high density and bulkiness have led to drawbacks such as increased fuel consumption, temporary loss of braking due to overheating, excessive wear, and brake fade, prompting researchers to explore lighter-weight alternatives such as composites^5^. Metal matrix composites (MMCs) have garnered significant attention over the past two decades due to their unique adjustable properties, making them highly desirable for industries like automotive and aerospace^6,7^. Compared to other matrices, MMCs exhibit substantial improvements in mechanical strength, enabling them to withstand compressive and tensile pressures. This attribute is achieved through the transmission and distribution of applied force from the reinforcement phase to the ductile matrix^6^. Ceramic particles are the most common reinforcements used in MMCs and have been shown to significantly enhance their mechanical properties, particularly when used as reinforcement for aluminum alloys^8^. However, the synthetic nature and cost of these ceramics have prompted researchers to seek cost-effective and abundant reinforcements with comparable or superior properties such as pumice and coal products^9^.

Pumice and coal ash are among such reinforcements being studied^10^. Pumice is a unique volcanic rock formed when lava rapidly cools, resulting in a highly porous and lightweight material. It contains a significant proportion of silicon dioxide (SiO_2_), aluminum oxide (Al_2_O_3_), and calcium oxide (CaO), rendering it a valuable component in various industries^11,12^. Similarly, coal ash is a unique material produced when coal is heated in an oven between temperatures 500 and 1300 °C in an air-deprived environment. It is composed of high amounts of graphite, SiO_2_, Al_2_O_3_, and Fe_2_O_3_, making it an excellent candidate for reinforcement in composite materials. The high graphite content of carbonated coal makes it an excellent material for use in brake disc applications, which serves as a solid lubricant, while its high strength and stiffness, provided by the SiO_2_, Al_2_O_3_, and Fe_2_O_3_ content, make it perfect for usage in a range of industrial applications^9,13–15^.

In addition to reinforcement’s type, size, geometry, and volume, the manufacturing route used to make composites of metal matrix also affects their attributes. The primary manufacturing route used to produce metal matrix composite is stir casting. Stir casting offers an economical method to produce MMCs. It involves dispersing reinforcing material particles into molten aluminum via mechanical stirring^16^. It can generate composites containing approximately 30% volume fraction of reinforcement material^16^. However, reinforcing particles tend to settle during solidification in the stir-casting process, leading to particle segregation. A two-step mixing process, also known as double stir casting, addresses some of the drawbacks of these conventional stir-casting techniques^17^. In this approach, the matrix material undergoes heating to about 30 °C beyond its liquidus point. Subsequently, the mixture is allowed to cool to a temperature range between the solidus and liquidus, achieving a semisolid state. Preheated reinforcements are introduced and thoroughly blended through vigorous mixing at this juncture. The resulting mixture is then reheated until it reaches a complete liquid state and stirs vigorously once more^18,19^. This approach results in a more homogeneous microstructure than traditional stirring, as a layer of gases around the particle’s surface is shattered, leading to better wetting of the molten metal and reinforcement particulates^19^, Peter and Adekunle^20^.

The quality and characteristics of the cast product are also greatly influenced by the process parameters utilized during casting. Monitoring and controlling these parameters influence the reaction synthesis that occurs during the processing of the composite melt. Overcoming these challenges, such as reinforcement distribution, wettability issues between the matrix and reinforced particles, porosity or gas trapping, and reaction viscosity, can accomplished by meticulously selecting and monitoring the process parameters utilized during casting. In the manufacture of aluminum matrix composites, various mechanical stirring process parameters have been employed^11,21^. The ultimate microstructure of the generated AMCs depends on these parameters to ensure that the reinforcements are uniformly distributed throughout the microstructure. Recent research has enhanced the Taguchi optimization technique’s output response by considering input factors influencing the technique’s performance metrics^11,22^.

This research explores how different process parameters (pouring temperature, stirrer speed, and stirring duration) and reinforcements’ volume fraction influence the composite’s tensile properties. The findings of this investigation will offer valuable perspectives on the production and optimization of hybrid composites for brake disc applications, helping to develop high-performance and cost-effective materials for the automotive industry. The study utilized the Taguchi optimization technique to obtain the optimal volume percentage of reinforcements and the process parameters of stir casting on the tensile strength of produced hybrid composites for brake disc production. The Taguchi optimization approach is a powerful statistical method extensively utilized to optimize process parameters and enhance product quality.

## Materials and methods

### Materials

The materials used to produce the Al-BP-CA hybrid composite are coal ash, brown pumice, and aluminum alloy (AA6061). The coal was acquired from a coal mine in Effeche-Akpalli, Benue State, Nigeria. The brown pumice was also extracted from an underground mining site in Biu, Borno State, Nigeria.

### Brown pumice particulates production process

The brown pumice was washed and dried for 48 h at 100 °C to remove filth and moisture. Respectively. The aggregated were pulverized with a laboratory mortar and pestle before being processed into powders using a ball milling machine. This manufacturing method aligns with studies by Ibrahim et al.^10^ and Jayakrishnan and Ramesan^23^. The brown pumice powder was further sieved per BSI 377:1990 standard into three different particle sizes (90, 56, and 25 μm).

### Production of coal ash particulates

The coals were initially washed and dried for 48 h at 100 °C to remove moisture and filth before pulverized it employing a jaw crusher. The pulverized pieces were put in a crucible made of graphite and heated in an electrical furnace without any air for 8 h to a heating temperature of 1100 °C. After being normalized in the oven, it was processed into powders using a ball milling machine. This method is supported by research by Hassan and Gomes^24^ and Sharma et al.^25^. The generated carbonized coal underwent further sieving per BSI 377:1990 standard to produce carbonized coal ash particles 25, 53, and 90 μm particle size.

### Experimental design

Taguchi’s design methodology was used to plan experimental runs. In this study, five factors: brown pumice particle (BP) (wt%), coal ash particles (CA) (wt%), stirrer speed (SP) (rpm), stirring duration (SD) (min), pouring temperature (°C), and four different levels were considered to be studied as shown in Table 1. The reinforcement, stirrer speed, pouring temperature, and stirring duration limits and levels were selected, referencing previous studies conducted by^26–28^. Minitab 21 software was used to generate the L16 orthogonal array for the 16 experimental trials, shown in Table 2.

**Table 1:** Reinforcements and stir casting process parameters factor and levels.

S/N	Processing factors	Unit	Factors designation	Level			
				1	2	3	4
1	Brown pumice	wt%	BP	2.5	5	7.5	10
2	Coal ash	wt%	CA	2.5	5	7.5	10
3	Stirrer speed	rpm	SS	200	300	400	500
4	Pouring temperature	°C	TP	700	750	800	850
5	Stirring duration	min	SD	5	10	15	20

**Table 2:** L_16_ Orthogonal array for production of hybrid composite.

Experimental run	Factors				
	BP (vol%)	CA (vol%)	SS (rpm)	TP (°C)	SD (min)
1	2.5	2.5	200	700	5
2	2.5	5	300	750	10
3	2.5	7.5	400	800	15
4	2.5	10	500	850	20
5	5	2.5	300	800	20
6	5	5	200	850	15
7	5	7.5	500	700	10
8	5	10	400	750	5
9	7.5	2.5	400	850	10
10	7.5	5	500	800	5
11	7.5	7.5	200	750	20
12	7.5	10	300	700	15
13	10	2.5	500	750	15
14	10	5	400	700	20
15	10	7.5	300	850	5
16	10	10	200	800	10

### Al-BP-CA hybrid composites fabrication

The composites were produced using a double-stir gravity casting procedure described by Ikubanni et al.^29^ and Adediran et al.^30^ methods using a bottom pouring stir casting machine at SwamEquip, Chennai, India.

Prior to the casting process, the brown pumice and coal ash particles underwent a preheating treatment for 2 h at 500 °C based on the recommendation of previous studies by Kumar et al.^31^, Madhukar et al.^32^ and Adebisi et al.^33^ to oxidize and calcine the particle surfaces. Subsequently, the aluminum alloys (AA6061) were fed into the electric furnace and heated to 800 °C, exceeding its liquidus temperature (660 °C) to guarantee thorough alloy melting. Before incorporating the preheated reinforcements, the surface dross was initially eliminated. Afterward, 0.01% NaCl (to eliminate gases) and 1% magnesium powders (to enhance wettability) were introduced into the molten aluminum^34^. The liquid alloy was permitted to undergo controlled cooling within the furnace until it reached a temperature of 610 °C, transitioning into a semisolid state. Subsequently, an automated stainless-steel stirrer coated with a protective layer was lowered into the furnace to initiate stirring to form a vortex within the melt. The preheated particulate materials were introduced gradually into the molten slurry at a steady flow rate of 5 g per minute^35^. Subsequently, the mixture was reheated to the prescribed pouring temperature, stirrer speed, and time as specified in the experimental run of the design plan. Prior to pouring the slurry into the mold, it was heated to approximately 450 °C^35^.

For each of the experimental trials, this methodology was adhered to, with careful consideration of the process parameters and the reinforcement percentage as recommended in the design plan^26^. A control (without reinforcement) was also produced to compare the effects of the reinforcement.

### Characterization of the constituents

Both matrix (aluminum AA6061) and reinforcement (pumice and carbonated coal) were characterized using XRF, SEM–EDS, XRD, and TGA/DTA to determine their chemical composition, morphology, crystalline structure, and thermal stability.

### Tensile strength (TS) test

**Figure 1:**
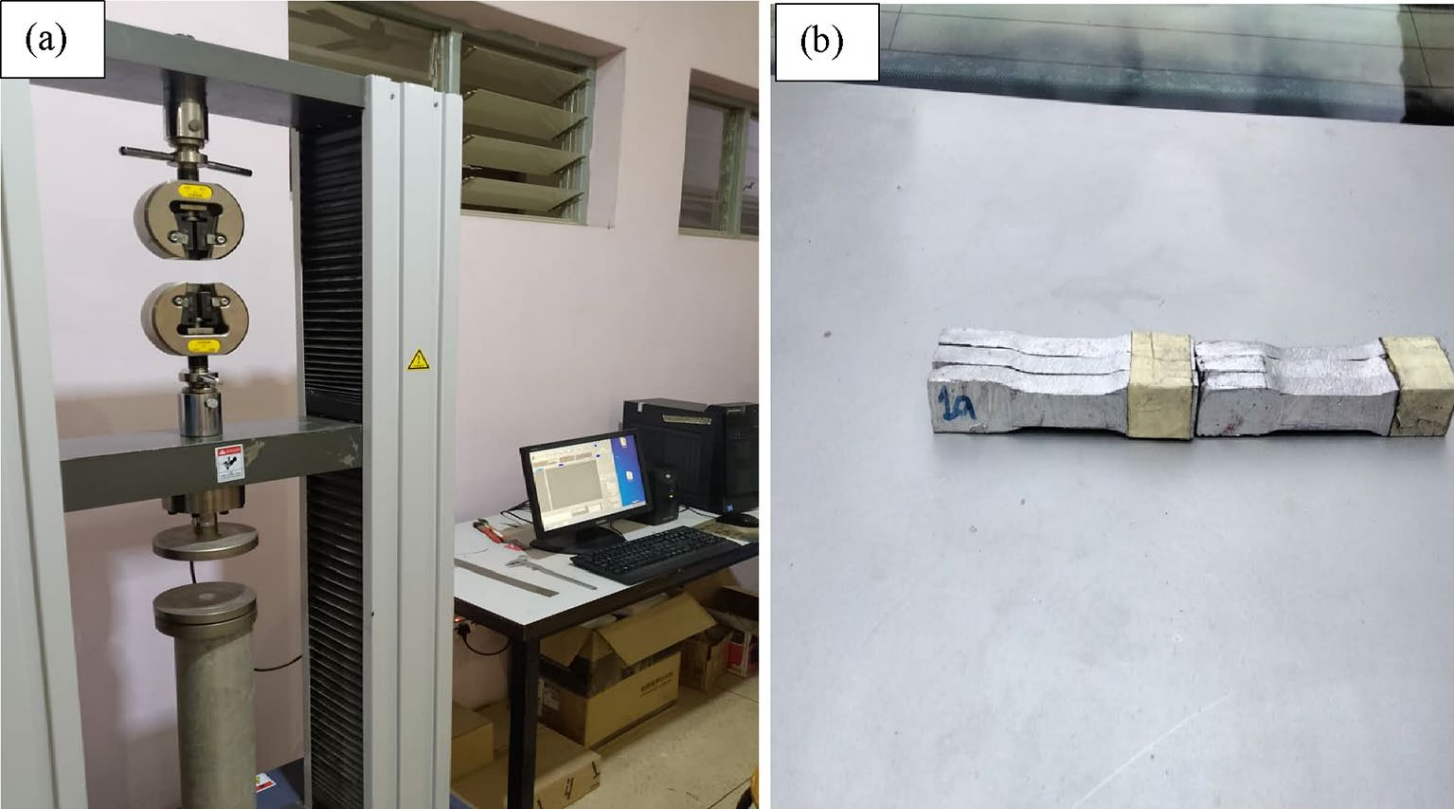
Experimental setup for tensile strength and the test samples produced.

The tensile test was conducted as per ASTM standards (E8M-09). The test samples were initially cut to dimensions of 100 × 10 × 6 mm, with a gauge length of 50 mm and a width of 6 mm. The test was carried out using a Computer Control Electronic Universal Testing Machine (WDW-1000KN) at room temperature with a cross-head test speed of 1 mm/min. To ensure data consistency and reliability, three repetitions of the tests were performed for each hybrid composite run^36^. The experimental setup for the test and the test samples produced are depicted in Figs. 1a and b, respectively.

### Statistical analysis and optimization

The Al-BP-CA hybrid composites’ tensile strengths were analyzed experimentally using Taguchi optimization and ANOVA with the aid of Minitab software (21.4.2). The study employed a “larger-the-better” objective function in Taguchi optimization to optimize the process parameters that can give us the best composite’s tensile strength^37^.

#### Confidence interval (CI)

The confidence interval for this analysis was evaluated using Eq. (1).

1 $$C.I. = \sqrt {f_{{ \propto \left( {1,d_{e} } \right)}} v_{e} \left( {\frac{1}{m} + \frac{1}{n}} \right)}$$

where $f_{{ \propto \left( {1, d_{e} } \right)}}$ is the F Distribution Critical Values (α = 0.05 significance level) between 1 and $d_{e}$ (which is the degree of freedom of error), gotten from statistical tables, $v_{e}$ is the variance (mean square) of error, which are obtained from the analysis of variance. N is the number of effective replications. M was evaluated using Eq. (2).

2 $$M = \frac{Total\, number\, of\, experiment}{{1 + degree\, of\, freedom\, of\, control\, factors}}$$

## Results and discussion

### X-ray fluorescence of the constituting materials

The constituents (aluminum alloy (AA6061), brown pumice, and coal ash particles) of the Al-BP-CA hybrid composites were examined via an XRF analyzer. Tables 3 and 4 present the results of the investigation. The XRF results of the aluminum alloy, as presented in Table 3, indicate that aluminum, silicon, and magnesium are the predominant constituents. This finding corroborates the findings of Kareem et al.^38^ and Ibrahim et al.^11^.

**Table 3:** Aluminium’s chemical composition.

Element	Al	Si	Fe	Cu	Mn	Mg	Zn	Cr	Ti	Ca	Others
vol%	97.743	0.61	0.44	0.16	0.024	0.821	0.0015	0.07	0.01	0.046	0.0745

**Table 4:** BP and CA chemical composition.

S/n	Elements	Brown pumice (%)	Coal ash particles (%)
1	O	42.267	46.52
2	Al	7.907	7.556
3	Si	20.594	22.141
4	P	0.288	0.000
5	S	0.074	4.415
6	Cl	0.718	1.721
7	K	4.212	1.130
8	Ca	8.574	2.667
9	Ti	2.140	3.500
10	V	0.084	0.140
11	Cr	0.008	0.170
12	Mn	0.198	0.268
13	Fe	12.348	8.910
14	Co	0.070	0.062
15	Ni	0.010	0.066
16	Cu	0.046	0.147
17	Zn	0.029	0.014
18	Zr	0.157	0.322
19	Nb	0.050	0.054
20	Mo	0.002	0.011
21	Ag	0.027	0.031
22	Ba	0.186	0.123
23	Ta	0.012	0.035

Table 4 shows the XRF results of the carbonated coal and pumice particles. The analysis verified that coal particle ash predominantly consisted of Si, Al, Fe, Ti, and Ca. The major constituents of pumice particles were Si, Fe, Al, Ca, K, and Ti. These findings align with the research conducted by Ibrahim et al.^10^, Arudiand Ozigi^39^, and Dagwa and Adama^40^. The presence of these elements indicates that brown pumice and coal ash particles have the potential to serve as effective particulate reinforcements in metal matrices given their chemical composition, which shares similarities with certain agricultural and industrial residue (bagasse, Coconut fiber ash, and Banana fibers ash, fly ash, bottom ash, and red mud)^40–43^, and^34^.

### Constituting materials’ X-ray powder diffraction

**Figure 2:**
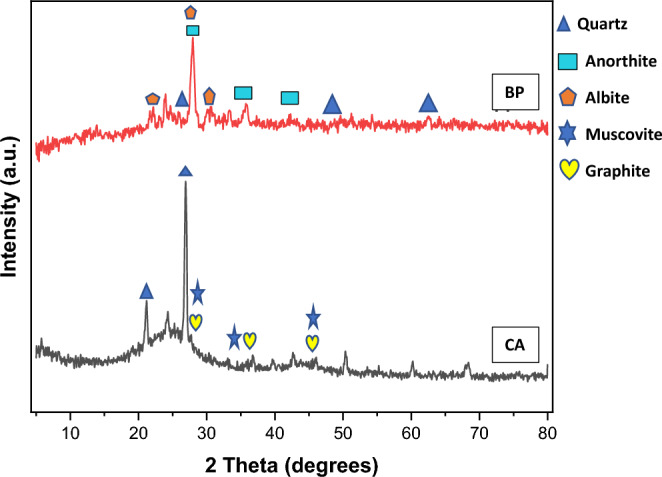
XRD results of BP and CA particles.

Based on the XRD patterns of brown particles (BP) displayed in Fig. 1, it is evident that the patterns include peaks corresponding to an amorphous quartz (SiO_2_) material, along with specific crystalline phases of anorthite (Ca Al_2_(SiO_4_)_2_) and albite (NaAlSi_3_O_8_). These findings correlate with Ersoy et al.^44^ and Pinarci and Kocak^45^ findings. Upon matching the coal ash particles, distinct phases of quartz (SiO_2_), graphite(C), and muscovite (KAl_2_(AlSi_3_O_10_)(F,OH)_2_) were identified, as shown in Fig. 2. This observation aligns with the research conducted by Ibrahim et al.^13^, who also identified some of these compounds in coal ash.

### Thermogravimetric and derivatives of thermal analysis of aluminum alloy

**Figure 3:**
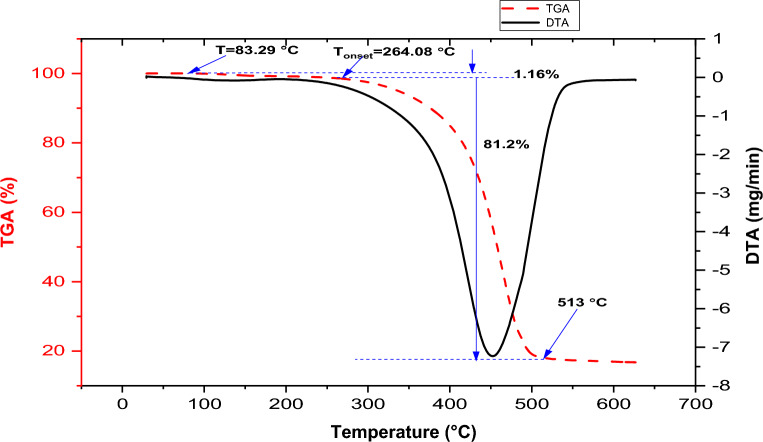
TGA-DTA curves of the aluminum alloy (AA6061).

The TGA-DTA curves in Fig. 3 demonstrate a two-step weight loss for aluminum alloy upon heating between 30 and 1000 °C under a nitrogen gas environment. The TGA curve in the figure shows a significant drop until it becomes parallel to the temperature axis at approximately 513 °C.

The initial 1.16% weight loss occurs between a temperature of 83.29 and 264.08 °C, which can be linked to the evaporation of the absorbed surface moisture and some volatile matter. The major decompositions of the materials occur in one stage between the temperatures of 264.08 and 513 °C with a mass loss of 81.2%.

### Thermogravimetric and derivatives of thermal analysis of carbonated coal particles

**Figure 4:**
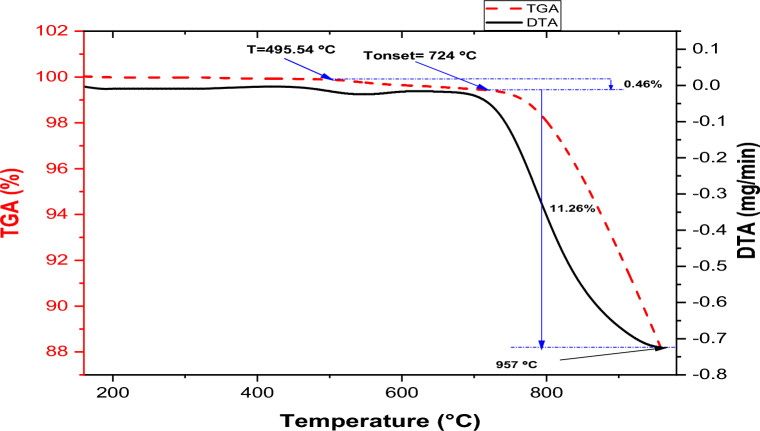
TGA-DTA curves of the pumice particle.

The TGA-DTA curves in Fig. 4 depict a two-step weight loss pattern for pumice particles when heating between 30 and 1000 °C under a nitrogen gas environment. The TGA curve in the figure demonstrates a substantial decline until it aligns parallel to the temperature axis around 957 °C.

The initial 0.46% weight loss occurs between a temperature of 495.54 and 724 °C was observed, which can be linked to the evaporation of the absorbed surface moisture and some volatile matters. The major decompositions of the materials occur in one stage between 724 and 957 °C with a mass loss of 11.26%. The major decompositions in pumice can be attributed to the thermal decomposition of volatile matters such as sulfur dioxide (SO_2_), carbon dioxide (CO_2_), nitrogen, and various trace gases that may be present in the volcanic environment during the rock’s formation. The lower mass loss in pumice particles is owing to its high melting points (1343 °C) and the presence of TiO_2_ (1843 °C) and SiO_2_ (1710 °C), the major constituents, compared to the lower melting temperature of the aluminum alloy. This observation is similar to the work of Gencel^46^.

### Thermogravimetric and derivatives of thermal analysis of carbonated coal particles

**Figure 5:**
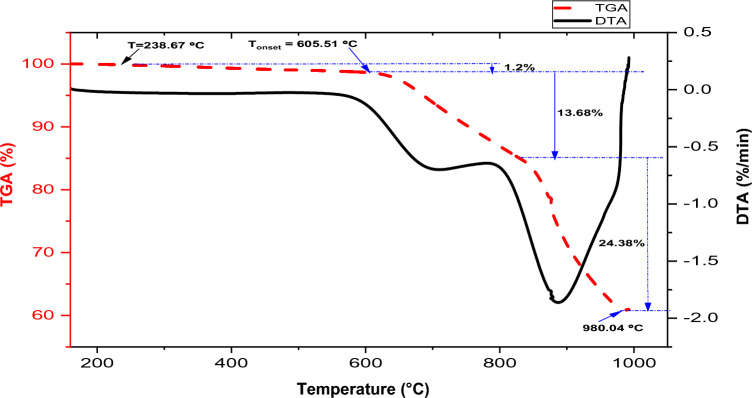
TGA-DTA curves of the carbonated coal particle.

The TGA-DTG curves in Fig. 5 demonstrate a three-step weight loss for carbonated coal particles upon heating between 30 and 1000 °C under a nitrogen gas environment. The TGA curve in the figure shows a significant drop until it becomes parallel to the temperature axis at approximately 980.04 °C.

The initial 1.2% weight loss occurs between a temperature of 238.67 and 605.51 °C was observed, which can be linked to the evaporation of the absorbed surface moisture and volatile matter. The major decompositions of carbonated coal particles occur in two stages: the first stage was between 605.51 and 840.52 °C with a mass loss of 13.68%, while the second was between 840 and 980.4 °C with a mass loss of 24.38%. The first stage of major decomposition in carbonated coal particles can be attributed to the volatile components such as water, hydrocarbons, or other gases trapped within the coal particles. The second stage might be due to the decomposition of carbonated minerals into oxides, which release carbon dioxide (CO_2_) gas. The reduced mass loss in carbonated coal particles is attributed to the elevated melting points of their primary constituents, TiO_2_ and SiO_2_ (1843 and 1710 °C, respectively), in comparison to the lower melting temperature of the aluminum alloy. These results agree with the findings of Omiogbemi et al*.*^47^.

### Tensile strength results and optimization of process parameters of stir casting

Table 5 displays the results of a tensile strength test performed on sixteen hybrid composites and a control sample, along with their signal-to-noise ratios. From the Table, the highest tensile strength was 169.09 MPa, which was recorded at run No.9. Whereas the lowest was 125.52 MPa, which was recorded at run No. 8. The Table further shows that the tensile strength exhibited by the as-cast aluminum alloy was 124.24 MPa. The increase in strength may stem from the inclusion of hard silica, anorthite, and albite in the reinforcements (as shown in the XRD analysis) and also the high thermal mismatch between the aluminum matrix (which has a greater thermal expansion coefficient (CTE)) and the reinforcement (lower thermal expansion coefficient). It has been proved that incorporating hard ceramic particles into the ductile aluminum matrix and thermal mismatched increases the strength of the composite. This result agrees with some related research conducted by Akinwande et al.^48^, and Zawrah et al.^49^, their studies show that the incorporation of the hard phase into the soft aluminum metal matrix always increases the tensile strength but reduces the ductility of the produced aluminum matrix composite.

**Table 5:** Tensile strength results and the signal to noise ratio of the Al-BP-CA Hybrid composite.

Runs	Factors					Tensile strength		
(S/N)	BP (vol%)	CA (vol%)	SP (rpm)	TP (°C)	SD (min)	Mean (MPa)	S/N ratio (dB)	Error bars
1	2.5	2.5	200	700	5	132.05	42.41	0.047
2	2.5	5	300	750	10	135.02	42.61	0.049
3	2.5	7.5	400	800	15	141.98	43.11	0.026
4	2.5	10	500	850	20	134.88	42.66	0.063
5	5	2.5	300	800	20	145.00	43.23	0.033
6	5	5	200	850	15	157.00	43.92	0.075
7	5	7.5	500	700	10	161.55	44.22	0.136
8	5	10	400	750	5	125.52	42.04	0.214
9	7.5	2.5	400	850	10	169.09	44.56	0.061
10	7.5	5	500	800	5	166.00	44.4	0.136
11	7.5	7.5	200	750	20	165.04	44.35	0.052
12	7.5	10	300	700	15	154.52	43.84	0.133
13	10	2.5	500	750	15	142.00	43.05	0.035
14	10	5	400	700	20	163.68	44.33	0.077
15	10	7.5	300	850	5	137.80	42.85	0.068
16	10	10	200	800	10	132.04	42.41	0.067
Mean						147.70	43.37	
Control (As Cast)						125.24		

#### Taguchi optimization of reinforcements casting process parameters

The higher-the-better objective function was used to observe the optimum and the most influential process parameters in the casting of aluminum hybrid composite. This analysis gave signal-to-noise ratios, main effect plots for the mean of the response, and mean of signal-to-noise ratios, as presented in the discussions below;

#### General mean and signal-to-noise ratio of the tensile strength

Table 5 shows 147.70 MPa and 43.37 dB as the sixteen runs’ average tensile strength and signal-to-noise ratio.

#### Effect of process parameters (stir casting) and reinforcements on the tensile strength

**Figure 6:**
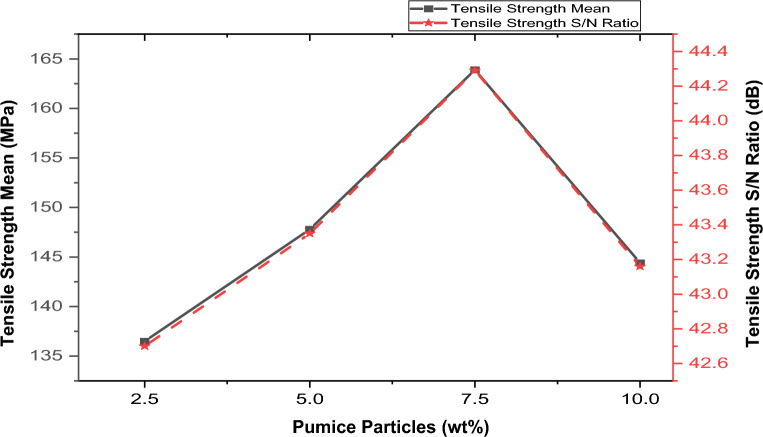
Variation of Brown pumice particle Content with Tensile Strength and S/N.

**Figure 7:**
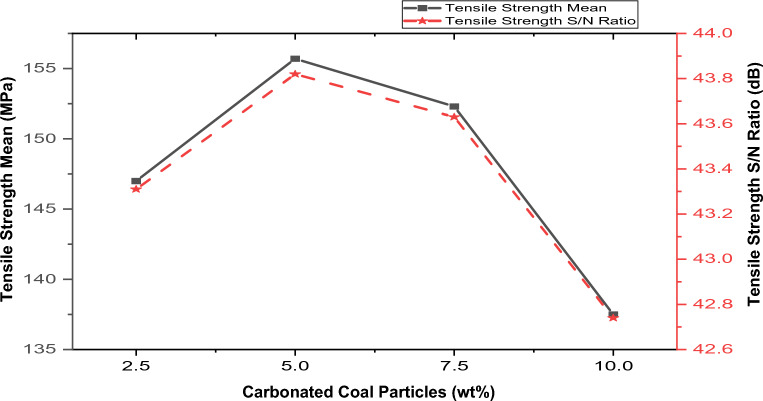
Variation of coal ash content with hybrid composite’s tensile strength and S/N.

**Figure 8:**
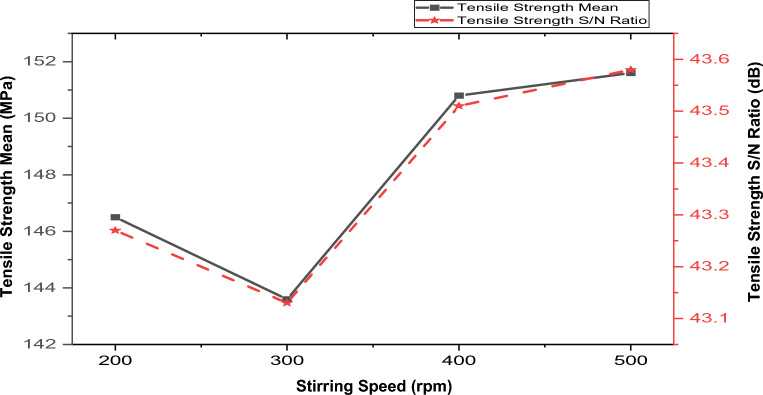
Variation of stirrer speed with hybrid composite’s tensile strength and S/N.

**Figure 9:**
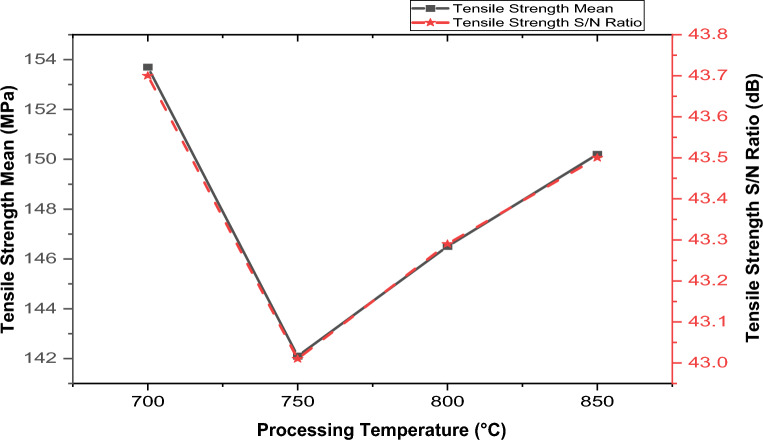
Variation of pouring temperature with hybrid composite’s tensile strength.

Figures 6, 7, 8 and 9 illustrate the variation of brown pumice particles, coal ash particles, stirrer speed, pouring temperature, and stirring time on tensile strength.

##### Effect of brown pumice particles on tensile strength

Figure 6 depicts the material’s tensile strength with varying compositions by volume percent of the pumice. The tensile strength exhibited an upward trend with increasing content of brown pumice particles, reaching its peak performance at 7.5% with a value of 163.9 MPa. However, beyond this threshold, additional reinforcement (exceeding 7.5 wt% pumice) led to a decrement in strength. The increase in tensile strength can be ascribed to adding pumice particulates (which contain quartz, anorthite, and albite, which are hard and stiff, as shown in XRD analysis) into the softer aluminum matrix. Also, it can be ascribed to the thermal mismatch between the aluminum matrix (high CTE) and the brown pumice particles (lower CTE) during the cooling and solidification of the composite). The decrease in strength can be ascribed to the reduction of wettability and the clustering (due to the interaction between the particles) of the pumice in the composite. According to Singh et al.^50^, clustering the reinforcement in the composite reduces its strength since it serves as a location for damage accumulation and fracture initiation. This finding is similar to the studies carried out by Aigbodion^34^, Hassan and Aigbodion^51^, and Reddy et al.^52^. According to their findings, increasing the reinforcement volume fraction enhanced the tensile strength of the aluminum composite to its maximum,however, after this point, the tensile strength declined with additional reinforcement. The analysis observed an optimal tensile strength of 163.9 MPa at 7.5 vol%.

##### Effect of coal ash particles on tensile strength

Figure 7 shows the material’s tensile strength with varying compositions by volume percent of the coal ash particles. The tensile strength increased as the concentration of coal ash increased to the highest performance of 155.7 MPa at 5%; beyond this point, further reinforcement addition led to a decrement in tensile strength. The increase in tensile strength can be ascribed to adding coal ash reinforcement (which contains quartz and muscovite, which are hard and stiff, as shown in XRD analysis) into the softer aluminum matrix. Also, the improvement in the strength can be ascribed to the thermal mismatch between the aluminum matrix (high CTE) and the brown pumice particles (lower CTE) during cooling and solidification of the composite). during cooling and solidification of the composite. The reduction in strength can be credited to reducing wettability and clustering of the coal ash in the composite. According to Singh et al.^50^, clustering of the reinforcements in the composite has a detrimental influence on the composite’s strength since it used to be a location for damage accumulation (local particle-rich zones are the most suitable nucleation sites for crack initiation). This finding is similar to the studies carried out by Aigbodion^34^, Hassan and Aigbodion^51^, and Reddy et al.^52^, according to the result of their findings, increasing the reinforcement content enhanced the tensile strength of aluminum composite to its maximum; however, after the optimum point, the tensile strength declined with additional reinforcement. The analysis observed an optimal tensile strength of 155.7 MPa at 5 vol%.

##### Effect of stirrer speed on tensile strength

Figure 8 shows increased tensile strength with increased stirrer speed during the composite production. Although there was a slight drop in tensile strength within 200–300 rpm stirrer speed, this phenomenon may be credited to the uneven reinforcement dispersion in the matrix at a lower speed. Increasing the stirrer speed generally had a favorable impact on the material’s tensile strength. Enhancements in tensile strength might stem from efficient reinforcements dispersion within the matrix before solidification. This behavior agrees with Malau et al.^53^ and Khosravi et al.^54^, whose Studies have shown that the aluminum composites’ tensile strength is contingent upon the reinforcement’s distribution within the matrix. The analysis observed the optimal tensile strength of 151.6 MPa at 400 rpm.

##### Effect of pouring temperature on tensile strength

According to the data presented in Fig. 9, changes in pouring temperature have a noticeable effect on the material’s tensile strength. Initially, as the pouring temperature increases from 700 to 750, there is a decrease in tensile strength. This decline could be ascribed to insufficient reinforcement dispersion, possibly owing to the high viscosity of molten aluminum, resulting in poor wettability. However, beyond the 750-degree threshold, there is a reversal in the trend, with tensile strength increasing as the temperature rises further. This observed increase might be attributed to the refinement of microstructural grains and a closer arrangement of grain boundaries. These changes could occur due to decreased melt viscosity at higher temperatures, leading to an enhanced tensile strength. The decrease in viscosity decreases the contact angle between the melted matrix and the reinforcements, thereby enhancing the distribution and wettability of the reinforcement in the matrix. This study agrees with Ibrahim et al.^11^, Hoff et al.^55^, and Jayashree et al.^56^, according to the results of their findings, they reported that increasing the melting temperature decreases the contact angle between the melted aluminum on the surface of the particles. From the analysis, the optimal tensile strength of 153.7 MPa was observed at 700 °C.

##### Stirring duration effect on the tensile strength

**Figure 10:**
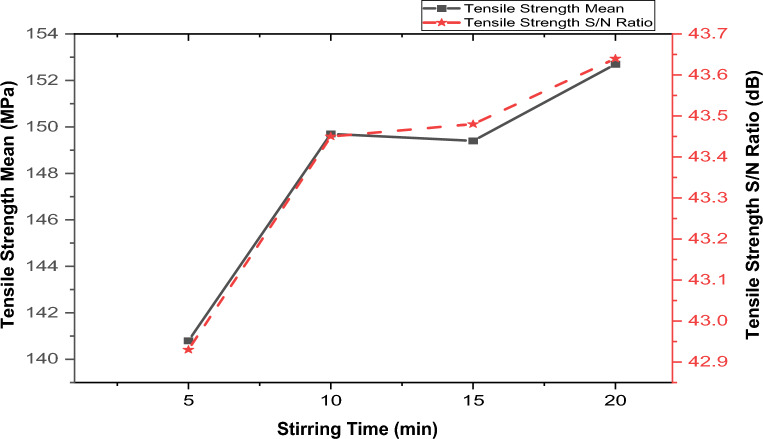
Variation of stirring time with tensile strength.

Figure 10 depicts the impact of stirring duration on the aluminum composite’s tensile strength. It reveals a positive correlation between tensile strength and stirring time during casting. This increase is likely accredited to the uniform dispersion of reinforcement within the matrix, facilitated by an appropriate stirring duration. This finding corroborates studies by Akinwande et al.^48^, Malau et al.^53^, and Singh et al.^50^, which also reported that longer stirring times enhance the composite’s tensile strength. An optimal tensile strength of 152.7 MPa was achieved after 20 min of stirring, as shown in the figure.

##### The optimum combination for tensile strength of the hybrid composite

From Figs. 6, 7, 8, 9 and 10, the optimum reinforcements and process parameters of stir casting that gives the best tensile strength are brown pumice particle (BP), coal ash (CA), and stirrer speed (SP) pouring temperature (TP). Stirring time (SD) are at 7.5%wt (level 3), 5%vol (level 2), 500 rpm (level 4), 700 °C (level 1) and 20 min (level 4), respectively. Therefore, a predicted optimum tensile strength of 186.81 MPa was obtained using Eq. (2).

##### Confirmation test

**Figure 11:**
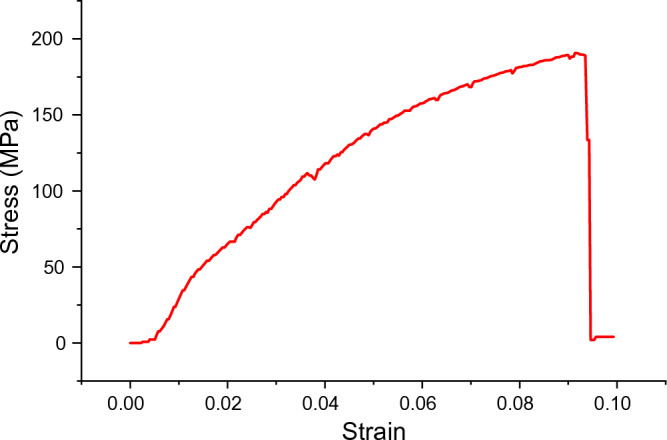
Stress/strain curve for optimal sample.

Table 6 shows the results of the tensile strength confirmation test. The analysis yielded an average tensile strength of 190.67 MPa. The stress/strain curve obtained from the optimal tensile test results is displayed in Fig. 11.

**Table 6:** Confirmation result of tensile strength test.

S/N	Factors					Tensile strength (MPa)			
	BP (vol%)	CA (vol%)	SP (rpm)	TP (°C)	SD (min)	Trial 1	Trial 2	Trial 3	Average
1	7.5	5	500	700	20	190.67	190.6	190.7	190.68

**Figure 12:**
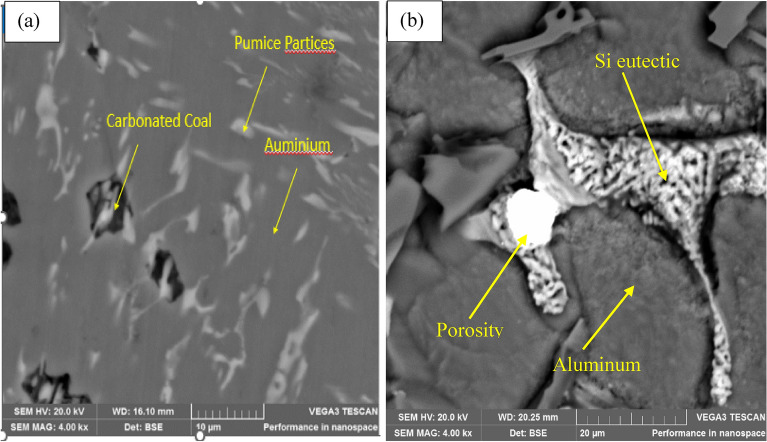
(**a**) SEM of the sample (**b**) SEM of the control sample.

Figure 12a and b showcase the scanning electron microscopy (SEM) results of the optimal and control samples, respectively. From the figure the grey color portion represent the aluminum alloy, whereas the white dendrites and the dark color portion represent the Si- eutectoid phases along with brown pumice and coal respectively as posited by Mourad et al.^57^. Interestingly, the optimal sample has a significantly finer grain structure than the control sample, which in turn may have contributed to the higher tensile strength observed in the former (190.68 MPa) compared to the latter (125.24 MPa).

Table 7 compares the tensile strengths of the developed aluminum composite with other researchers for producing automobile brake discs. The developed brake disc shows an improvement of 92, 1.4, and 101% compared to the works of Rong^4^, Nong et al.^58^, and Anand et al.^59^, respectively. However, there is a 27% reduction compared to the work of Hong et al.^60^. Despite this reduction, the produced brake disc meets the minimum requirement of 150 MPa, as stipulated by Rong^4^.

**Table 7:** Comparison of tensile strength of MMC for brake disc application.

Materials	Tensile strength (MPa)
Aluminium Alloy 2024 + 3 wt SiC	264 ^60^
Aluminium Alloy + 5 wt% Al_2_O_3_	99.33 ^4^
SiC3D/Al alloy	188 ^58^
10 vol% Cenosphere-AA6063	96 ^59^
Aluminium Alloy 6061 + 7.5 wt% Brown Pumice + 5 wt% Coal ash	190.56 (Present work)

Table 8 shows the results of the predicted and the experimental tensile strength of optimal processing parameters (BP_3_–CA_2_–SP_4_–TP_1_–SD_4_) of the hybrid aluminum matrix composite, which gives an experimental error of 2.02%, which is within the acceptable limit in engineering^61^.

**Table 8:** Predicted and experimental tensile strength.

	Optimum parameter settings	Predicted value (MPa)	Experimental value (MPa)	% error
S/N (dB)	BP_3_–CA_2_–SP_4_–TP_1_–SD_4_	45.43	45.61	0.39
TS (MPa)		186.81	190.67	2.02

### Regression analysis (modeling)

Table 9 shows the ANOVA table obtained from the produced hybrid composite’s tensile strength results. At 0.05 significant level, the regression model and all the factors are significant, except coal ash particles, which have a *p*-value greater than 5%.

**Table 9:** ANOVA table of tensile strength of hybrid composite.

Source	DF	Adj SS	Adj MS	F-Value	*P*-Value	% contribution
Regression	13	3127.04	240.54	23,868.48	0.00	
Brown Pumice (BP)	1	694.51	694.51	68,914.55	0.00	15.20
Coal Ash (CA)	1	0.01	0.01	1.29	0.37	0.00
Stirer Speed (SP)	1	108.39	108.39	10,755.17	0.00	2.37
Pouring temperature (TP)	1	913.44	913.44	90,638.50	0.00	19.99
Stirring time (SD)	1	764.78	764.78	75,887.27	0.00	16.73
SP^2^	1	31.69	31.69	3144.37	0.00	0.69
TP^2^	1	910.81	910.81	90,378.15	0.00	19.93
SD^2^	1	828.58	828.58	82,218.73	0.00	18.13
CA_*_SP	1	7.89	7.90	783.39	0.00	0.17
CA_*_TP	1	78.30	78.30	7769.97	0.00	1.71
SP_*_TP	1	41.56	41.56	4124.07	0.00	0.91
CA_*_SP_*_TP	1	141.21	141.21	14,012.03	0.00	3.09
SP^2^_*_TP	1	39.25	39.25	3895.11	0.00	0.86
Error	2	10.02	0.01			0.22
Total	15	4570.44				100

Equation (3) shows the predictive model (mathematical) for the tensile strength (TS) as a function of the brown pumice, coal ash, stirrer speed, pouring temperature, and stirring duration.

3 $$\begin{aligned} TS = & \,8140.9 - 24.0205BP \\ & \; - 1.20CA + 3.4149SP \\ & \; - 21.8840TP - 37.824SD \\ & \; - 0.002980SP^{2} + 0.014498 TP^{2} \\ & \; + 1.57210SD^{2} + 0.08581CA*SP \\ & \; + 0.11779CA*TP - 0.002556 SP*TP \\ & \; - 0.000446CA*SP*TP + 0.000004 SP^{2} *TP \\ \end{aligned}$$

**Figure 13:**
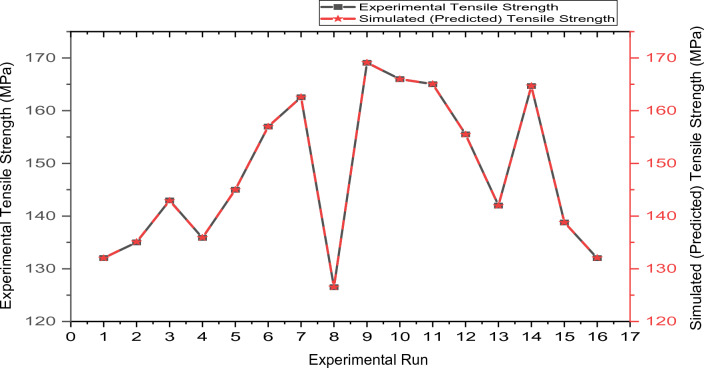
Experimental and predictive tensile strength.

The capability of the predictive mathematical model was evaluated employing the coefficient of determination, R-Square (R^2^), R-Square adjusted (R^2^-adj), and R-Square predicted (R^2^-pred). The developed regression model of the tensile strength has a high coefficient of determination values of 100%, 100%, and 99.93% for R^2^, R^2^-adj, and R^2^-pred, respectively. These values show a good fit between tensile strength values and the stir process parameters since they are almost 100%. According to Dan-Asabe et al.^62^ and Ibrahim et al.^11^, an R-Square value exceeding 75% is considered satisfactory. Figure 13 compares the tensile strength data generated using the model equation (Eq. 3) and the experimental values for the 16 different runs.

### Confidence interval (CI)

A confidence interval of ± 10.76 was evaluated using Eq. (1). The confirmatory tensile strength (190.67 MPa) obtained falls within the CI range of the tensile strength, indicating consistency with the predicted values as shown below:

$$\begin{gathered} {\text{TS}}_{{{\text{predictive}}}} {-}{\text{ CI}} < {\text{ TS}}_{{{\text{experimental}}}} < {\text{ TS}}_{{{\text{predictive}}}} + {\text{CI}} \hfill \\ {176}.0{5 } < {\text{ TS}}_{{{\text{experimental}}}} < { 197}.{57} \hfill \\ \end{gathered}$$

This outcome confirms the validity of the predicted optimum tensile strength value, as it falls within the 95% confidence interval range.

#### Thermal analysis of the optimal hybrid composite

**Figure 14:**
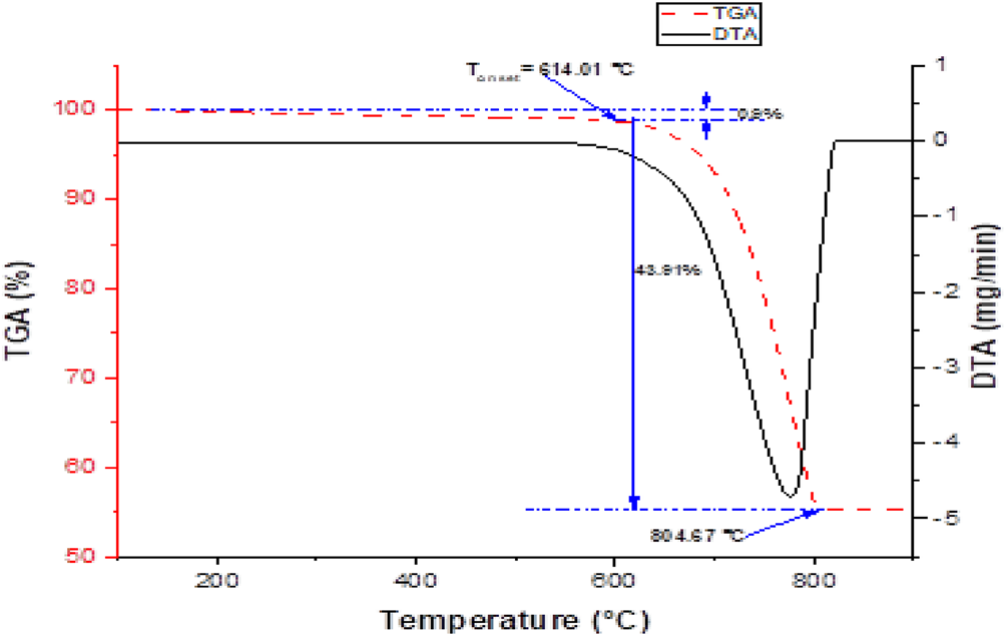
TGA-DTA curves showing thermal decompositions of optimal hybrid composite.

The thermo-gravimetric and differential thermo-gravimetric analyses (TGA/DTA) curves in Fig. 14 demonstrate a two-step weight loss for optimal hybrid composite upon heating between 30 and 1000 °C under a nitrogen gas environment.

The initial 0.9% weight loss observed in the figure can be attributed to the evaporation of absorbed surface moisture. The major decompositions of the materials occur in one stage between 614.01 and 804.7 °C, with a mass loss of 43.91%. The major mass loss in composites can be accredited to the thermal decomposition of volatile matter and the combustion of organic components in the composite. The figure shows the onset temperature of the optimal hybrid composite to be 614 °C. From the above analysis, we can conclude that pumice and carbonated coal enhanced the thermal resilience of the aluminum alloy composite. Therefore, the hybrid composite can withstand the maximum operating temperature of a lightweight vehicle (500 °C) that a disc can undergo during braking.

## Conclusion

The study considered the production, characterization, and optimization of the process parameters of stir casting and reinforcements (brown pumice and coal ash particles) on the tensile strength of the produced aluminum hybrid composites. The Al-BP-CA hybrid composite was also successfully produced and characterized. Based on the findings, the following conclusions can be drawn:

i. During the XRD characterization, SiO_2_, Fe_2_O_3_, and Al_2_O_3_ were discovered in the BP and CA as the major phases, making them acceptable for reinforcements in various metal matrixes. XRF characterization indicates that aluminum, silicon, and magnesium are the predominant constituents in the aluminum alloy (AA6061) used; the coal particle ash predominantly consisted of Si, Al, Fe, Ti, and Ca, while the major constituents of pumice particles were Si, Fe, Al, Ca, K, and Ti.
ii. The thermogravimetric analysis shows that the aluminum alloy (as-cast), brown pumice, and coal ash can withstand a temperature of 264.08, 724, and 605.51 °C.
iii. According to research that used stir casting process parameter optimization, the CA, SD, TP, and SP had less influence on the hybrid composite’s tensile strength than BP.
iv. The Taguchi optimization gave 7.5 vol% of brown pumice particulate, 2.5 vol% of coal ash, 700 °C pouring temperature, 500 rpm stirrer speed, and 20 min stirring duration as optimal process parameters for the tensile strength. The experimental and predictive tensile strengths were determined to be 190.66 and 186.81 MPa, respectively. These values represent an increase of 52.23% and 49.16% over the tensile strength of control. Furthermore, the resulting tensile strength from the confirmatory test confirms that the experimental tensile strength is within the CI range.
v. The regression analysis results show that BP has the highest contribution. Moreover, the predictive model (mathematical) developed for tensile strength as a function of BP, CA, SP, TP, and SD demonstrates a high level of prediction, with R-Square, R-Square adjusted, and R-Square predicted values of 100, 100, and 99.93%, respectively.
vi. The thermogravimetric analysis shows that the optimal composite can withstand a temperature of 614.01 °C, and adding brown pumice and coal ash improved the thermal degradation by 132.51%.

## Scope of feature work

Evaluating the tribological properties of the hybrid composite.

## Data Availability

The raw/processed data required to reproduce these findings cannot be shared at this time as the data also forms part of an ongoing study (PhD Thesis).
